# Differential Subcellular Distribution and Translocation of Seven 14-3-3 Isoforms in Response to EGF and During the Cell Cycle

**DOI:** 10.3390/ijms21010318

**Published:** 2020-01-02

**Authors:** Abdalla Abdrabou, Daniel Brandwein, Zhixiang Wang

**Affiliations:** Department of Medical Genetics, and Signal Transduction Research Group, Faculty of Medicine and Dentistry, University of Alberta, Edmonton, AB T6G 2H7, Canada; abdrabou@ualberta.ca (A.A.); brandwei@ualberta.ca (D.B.)

**Keywords:** 14-3-3 proteins, isoforms, subcellular localization, mitochondria, centrosome, nucleus, actin fibers, and microtubules

## Abstract

Multiple isoforms of 14-3-3 proteins exist in different organisms. In mammalian cells, 14-3-3 protein has seven isoforms (α/β, ε, η, γ, σ, θ/τ, and δ/ζ), with α and δ representing the phosphorylated versions of β and ζ, respectively. While the existence of multiple isoforms may represent one more level of regulation in 14-3-3 signaling, our knowledge regarding the isoform-specific functions of 14-3-3 proteins is very limited. Determination of the subcellular localization of the different 14-3-3 isoforms could give us important clues of their specific functions. In this study, by using indirect immunofluorescence, subcellular fractionation, and immunoblotting, we studied the subcellular localization of the total 14-3-3 protein and each of the seven 14-3-3 isoforms; their redistribution throughout the cell cycle; and their translocation in response to EGF in Cos-7 cells. We showed that 14-3-3 proteins are broadly distributed throughout the cell and associated with many subcellular structures/organelles, including the plasma membrane (PM), mitochondria, ER, nucleus, microtubules, and actin fibers. This broad distribution underlines the multiple functions identified for 14-3-3 proteins. The different isoforms of 14-3-3 proteins have distinctive subcellular localizations, which suggest their distinctive cellular functions. Most notably, 14-3-3ƞ is almost exclusively localized to the mitochondria, 14-3-3γ is only localized to the nucleus, and 14-3-3σ strongly and specifically associated with the centrosome during mitosis. We also examined the subcellular localization of the seven 14-3-3 isoforms in other cells, including HEK-293, MDA-MB-231, and MCF-7 cells, which largely confirmed our findings with Cos-7 cells.

## 1. Introduction

Protein phosphorylation is a central mechanism regulating cell signaling, and 14-3-3 proteins are important parts of this regulatory network, as they specifically bind to Ser and Thr phosphorylated proteins. The 14-3-3 family proteins comprise seven isoforms and exist as homo- and/or heterodimers in cells [1]. In mammalian cells, the r protein has seven isoforms (α/β, ε, γ, η, σ, θ/τ, and δ/ζ), with α and δ representing the phosphorylated versions of β and ζ, respectively [2,3,4,5,6]. Through interactions with Ser/Thr phosphorylated intracellular proteins, 14-3-3 proteins alter the activities, conformations, and subcellular localizations of their binding proteins [1,4,7,8]. The proteins binding to 14-3-3 proteins are involved in cytoskeleton remodeling, cell signaling, transcription regulation, and DNA repair. Thus, 14-3-3 proteins are able to regulate diverse cellular functions, including cell motility, cell cycle, cell proliferation, and apoptosis. Noticeably, many proteins involved in actin remodeling have recently been identified as 14-3-3 binding partners [5,6,7,9]. While the overexpression of 14-3-3 proteins has been implicated in many cancers, down-regulation of 14-3-3 expression plays a role in tumor suppression [10,11,12,13,14,15]. For example, several 14-3-3 protein isoforms, including β, γ, ε, ζ and θ, are highly expressed in lung cancer [16]. Many breast cancer patients show the overexpression of 14-3-3θ, and this overexpression is associated with a lower survival rate [17]. Particularly, 14-3-3τ promotes cell growth by stimulating ubiquitin-independent proteasome degradation of p21Waf1/Cip1 (the cyclin-dependent kinase inhibitor) [17]. Of all the 14-3-3 isoforms, 14-3-3σ and ζ have been most directly linked to cancer; however, they produce opposite effects [18]. 14-3-3σ induces cell cycle arrest at the G2-M transition to function as a tumor suppressor [19]. Expression of 14-3-3σ has been shown to be down-regulated in many cancers, including prostate [20], bladder [21], and ovarian cancers [22]. In contrast, 14-3-3ζ shows oncogenic effects. Its overexpression is linked to enhanced tumor growth. The inhibition of 14-3-3ζ has been a targeted therapeutic strategy in prostate cancer treatment [23,24].

14-3-3 proteins are evolutionarily conserved with molecular weights ranging from 28 to 33 kDa. The 14-3-3 proteins are widely expressed and bind to a large number of intracellular proteins in normal and cancer cells [25,26]. Two phosphorylation-dependent high-affinity binding motifs have been originally defined for binding 14-3-3 proteins: RSXpSXP (mode I) and RXY/FXpSXP (mode II) [27,28]. In addition, 14-3-3 proteins also bind to the extreme C-termini (pSX1–2–COOH) of numerous proteins. This motif was recently defined as mode III [6,29,30]. 

In mammalian cells, the 14-3-3 protein has seven isoforms and each isoform is encoded by a separate gene [1]. While the existence of multiple isoforms may represent one more level of regulation in 14-3-3 signaling, our knowledge regarding the isoform-specific functions of 14-3-3 proteins is very limited. The difficulty in studying the isoform-specific function is partly due to the numerous and varied functions proposed for these proteins.

Determination of the subcellular localization of the different 14-3-3 isoforms could give us important clues of their specific functions. While there are some limited studies regarding the subcellular localizations of 14-3-3 isoforms, they are mostly regarding the 14-3-3 proteins in yeast, flies, and plants [31,32]. It was previously reported that both 14-3-3γ and ε are associated with the centrosome in the human spleen [33]. It was recently shown that 14-3-3γ is localized to the centrosome and the loss of 14-3-3γ leads to centrosome amplification [34]. All brain isoforms of 14-3-3 were detected in the cytoplasmic compartment of the rat hippocampus, while 14-3-3γ and ζ were also present in mitochondrial and microsome-enriched fractions [35]. Initially, 14-3-3ζ was localized in the glial cell progenitor cytoplasm but translocated into the nucleus when the progenitor differentiated [36]. It was further reported that 14-3-3ζ regulates the nuclear trafficking of protein phosphatase 1α [37]. The 14-3-3θ isoform is also localized in both the cytoplasm and the nucleus and forms a ternary protein complex with serum- and glucocorticoid-induced protein kinase 1 and Tau, a microtubule-associated protein [38]. Tissue and cell-specific expressions of 14-3-3 isoforms have also been reported [36,39].

So far there has not been any comprehensive study to determine the subcellular localization of each of the seven 14-3-3 isoforms, their specific redistributions throughout the cell cycle, and their translocations in response to growth factors in the same cells/tissues. In this study, by using indirect immunofluorescence, subcellular fractionation, and immunoblotting, we studied the subcellular localization of the total 14-3-3 protein and each of the seven 14-3-3 isoforms, their redistribution throughout the cell cycle, and their translocation in response to EGF in Cos-7 cells. We showed that 14-3-3 isoforms have very different subcellular localization profiles, which could reflect their distinctive roles in regulating cell functions through interacting with a variety of other proteins.

## 2. Results

### 2.1. The Expression and Subcellular Localization of Total and Each Isoform of 14-3-3 Proteins

**Figure 1:**
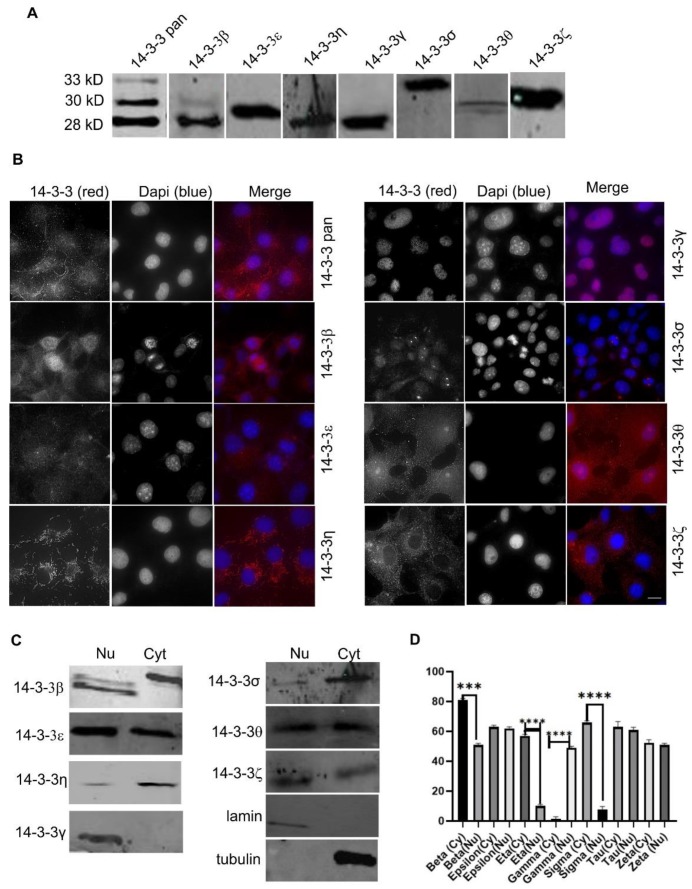
Subcellular localization of total 14-3-3 protein and the seven 14-3-3 isoforms in Cos-7 cells. The total 14-3-3 protein was determined by a pan14-3-3 antibody. Each 14-3-3 isoform was determined by antibodies to each specific isoform. Immunoblotting, immunofluorescence, and subcellular fractionation were performed as described in Materials and Methods. (**A**) The expression level of total protein and seven isoforms of 14-3-3 protein by immunoblotting in Cos-7 cells. The expression of tubulin was used as a control. (**B**) Subcellular localization of pan14-3-3 and seven 14-3-3 isoforms in Cos-7 cells by immunofluorescence. 14-3-3 proteins were revealed by TRITC (red) and the chromatin was stained by DAPI (blue). Scale bar = 10 µm. (**C**) Nuclear and cytoplasmic localization of 14-3-3 proteins by subcellular fractionation followed by immunoblotting. A-Tubulin was used as a marker for cytoplasm and lamin A was used as a marker for the nucleus. (**D**) Quantification of the results from (**C**). each value is the average of three experiments and the error bar is the standard error. **** *p* < 0.0001; *** *p* < 0.001.

We first determined the expression of the total 14-3-3 proteins (with a pan 14-3-3 protein antibody) and each of the seven 14-3-3 isoforms by immunoblotting in Cos-7 cells. As shown in Figure 1A, the pan 14-3-3 antibody detected three bands with molecular weights of 28, 30, and 33 kDa, respectively. Each of the seven 14-3-3 isoforms is readily detectable with antibodies specific to the isoforms. The molecular weights of 14-3-3β, ƞ, and γ correspond to the lower band of pan 14-3-3 at 28 kDa. The molecular weights of 14-3-3ε, θ, and ζ correspond to the middle band of pan 14-3-3 at 30 kDa. The molecular weight of 14-3-3σ corresponds to the higher band of pan 14-3-3 at 33 kDa. All of the blots for the isoforms only show one band, except for 14-3-3β. In the 14-3-3β blot, there is one strong band and the other band is very weak. However, this still suggests that our 14-3-3β antibody may have some weak interactions with another isoform.

We next examined the subcellular localization of total 14-3-3 and each 14-3-3 isoform by indirect immunofluorescence. As shown in Figure 1B, pan 14-3-3 was stained positive both in the nucleus and in the cytoplasm, and in the cell junctions. The most prominent cytoplasm stain of pan 14-3-3 is a fiber-like pattern both near the plasma membrane and across the cell, which resembles the actin fibers. In addition, pan 14-3-3 also stained positive throughout the cytoplasm. 14-3-3β showed both cytoplasmic and nuclear stains. We also observed certain weak microtubule-like patterns of 14-3-3β. 14-3-3ε is almost exclusively localized in the cytoplasm. In the cytoplasm, 14-3-3ε stains showed strong particles, mostly in the perinuclear region in one side of the nucleus, most likely associated with the Golgi apparatus. 14-3-3ƞ, γ, σ, and ζ all showed very specific stains, indicating their specific subcellular localizations. 14-3-3ƞ showed an almost exclusive mitochondrial pattern, which suggests that 14-3-3ƞ is solely localized to the mitochondria. 14-3-3γ was completely localized to the nucleus. 14-3-3γ formed fine particles throughout the nucleus but was absent from the nucleoli. In interphase cells, 14-3-3σ was stained positive both in the nucleus and in the cytoplasm. However, most strikingly, 14-3-3σ showed strong and specific centrosome staining during mitosis. 14-3-3θ is localized to both the nucleus and the cytoplasm but did not show specific associations with any organelle. 14-3-3ζ is exclusively localized to the cytoplasm, without any nuclear presence. In the cytoplasm, 14-3-3ζ showed some weak ER patterns and microtubule patterns. 

We further confirmed our immunofluorescence observations by subcellular fractionation and immunoblotting. We isolated nuclear fractions from the total cell homogenates. By using lamin A as the marker for the nucleus and α-tubulin as the marker for the cytoplasm, we showed that our fractionations are very specific (Figure 1C,D). As shown in Figure 1C,D, 14-3-3β, ε, θ, σ, and ζ were detectable in both the nuclear and the cytoplasmic fractions; 14-3-3γ was only detectable in the nuclear fraction; and 14-3-3ƞ and σ were primarily detected in the cytoplasmic fractions.

**Figure 2:**
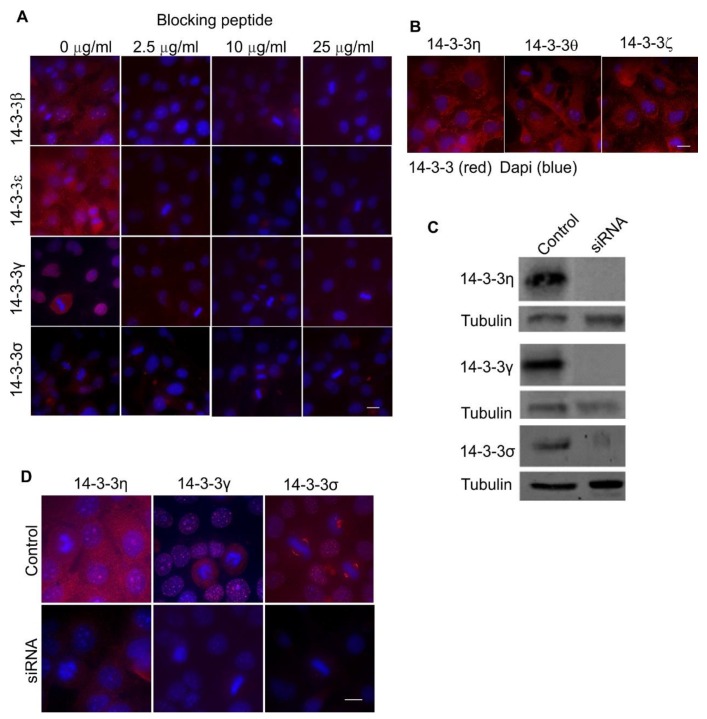
Control experiments to determine the specificities of the antibodies used in Figure 1 by indirect immunofluorescence in Cos-7 cells. (**A**) The effects of blocking peptides for 14-3-3β, ε, γ, and σ. The indirect immunofluorescence experiments were performed as described for Figure 1, except that the antibodies were incubated with blocking peptides of the indicated concentration for 1 h prior to incubation with the cells. (**B**) The subcellular localizations of 14-3-3η, θ, and ζ were determined by antibodies different from those used in Figure 1; (**C**) 14-3-3η, γ, and σ were knocked down in HEK 293 cells by siRNA, and the expressions of these isoforms were examined by immunoblotting with the corresponding antibodies. (**D**) 14-3-3η, γ, and σ were knocked down in HEK 293 cells by siRNA and the expressions of these isoforms were examined by indirect immunofluorescence with the corresponding antibodies. Scale bar = 10 µm.

It is important to test the specificities of the antibodies and to validate our observations. Among the antibodies to the seven 14-3-3 isoforms, four antibodies, including antibodies to 14-3-3β, ε, γ, and σ, are raised against short peptides. As such, we tested their specificity by using the peptides as blocking reagents. We showed that in a dose-dependent manner, these peptides specifically and effectively blocked the observed positive stains in IF (Figure 2A).

For three antibodies, including those for 14-3-3ƞ, θ, and ζ, there were no blocking peptides available. To validate our IF data, we performed the same experiments with different antibodies against those isoforms. As shown in Figure 2B, similar subcellular localizations of these 14-3-3 isoforms were revealed by these new antibodies.

Only three of the seven isoforms, including 14-3-3ƞ, γ, and σ showed very specific subcellular localizations to mitochondria, the nucleus, and the centrosome, respectively. We further examined the specificities of the three antibodies by siRNA knockdowns followed by immunoblotting and immunofluorescence. As shown in Figure 2C,D, when 14-3-3ƞ, γ, and σ were knocked down by siRNA in HEK 293 cells, the isoform-specific antibodies to 14-3-3ƞ, γ, and σ did not detect the corresponding 14-3-3 protein isoforms by immunoblotting (Figure 2C) or immunofluorescence (Figure 2D), which further validated the specificities of the antibodies.

### 2.2. Subcellular Localization and Translocation of Pan 14-3-3 in Response to EGF and During the Cell Cycle 

We next examined the subcellular localization and the translocation of the total 14-3-3 protein and each of the seven 14-3-3 isoforms in response to EGF and during the cell cycle by indirect immunofluorescence. We first studied the total 14-3-3 (pan14-3-3) protein in Cos-7 cells by using a pan 14-3-3 antibody.

**Figure 3:**
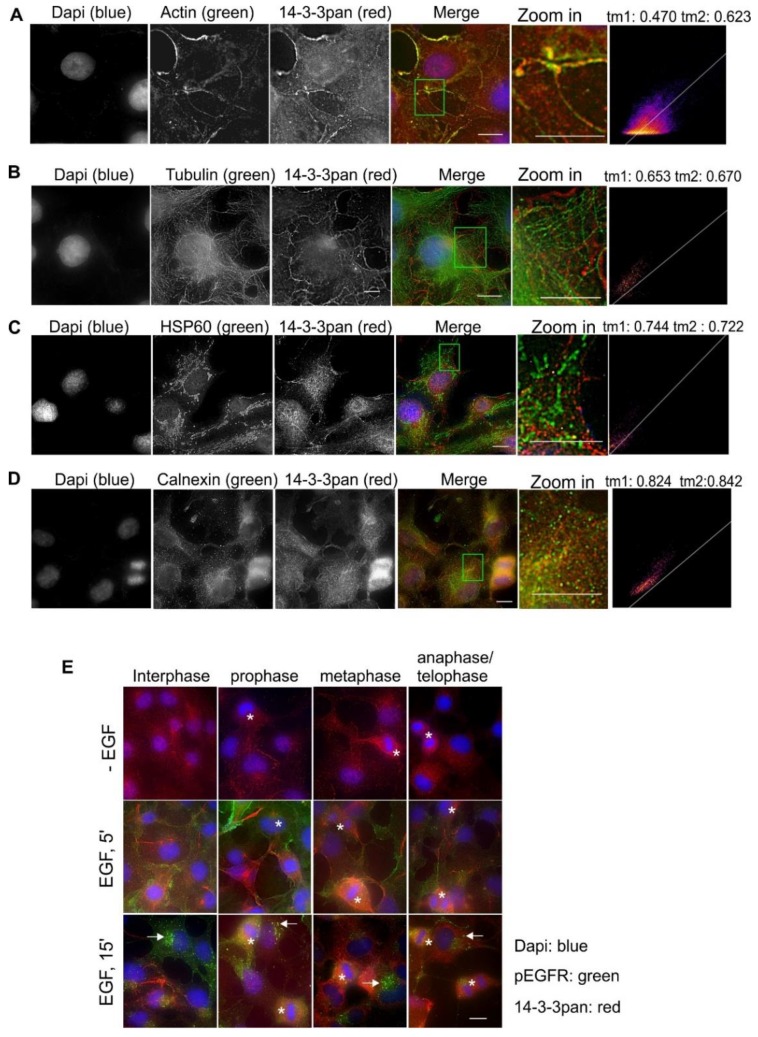
Subcellular localization of the pan14-3-3 protein in Cos-7 cells by double indirect immunofluorescence. (**A**) Co-localization of pan14-3-3 (red) and actin (green); (**B**) co-localization of pan14-3-3 protein (red) and tubulin (green); (**C**) co-localization of pan14-3-3 protein (red) and HSP60 (green); (**D**) co-localization of pan14-3-3 protein (red) and calnexin (green). For (**A**–**D**), the zoomed-in areas were used to calculate Mander’s coefficients. (**E**) Subcellular localization of pan14-3-3 protein during the cell cycle and in response to EGF. With or without EGF stimulation, pan14-3-3 proteins (red) and pEGFR (green) were revealed by double indirect immunofluorescence. Mitotic cells are labeled by * and endosomes are marked by arrows. Scale bar = 10 μm.

Based on the pattern of the subcellular distribution of pan14-3-3, we examined if pan14-3-3 co-localizes with various markers by immunofluorescence and quantitatively analyzed the co-localization by Mander’s coefficients. α-Actin and α-tubulin are the markers for the cytoskeleton, HSP60 is the marker for the mitochondria, calnexin is the marker for the endoplasmic reticulum (ER), and phosphorylated EGFR (pEGFR) is the marker for the plasma membrane (PM) and the endosomes. Shown in Figure 3, pan14-3-3 strongly co-localized with actin fibers, especially in the regions close to the cell membrane, possibly lamellipodium (Figure 3A). Pan14-3-3 showed very limited co-localization with tubulin (Figure 3B) and HSP60 (Figure 3C), but noticeable co-localization with calnexin (Figure 3D). The localization of pan14-3-3 in both the nucleus and the cytosol was also prominent. 

We then examined whether EGF stimulates the re-distribution of pan14-3-3 and whether the subcellular localization of pan14-3-3 changed during mitosis. As shown in Figure 3E, EGF seemed to increase the localization of pan14-3-3 to the fiber structures, likely actin, as revealed above in Figure 3A. Similarly, the fiber-like stain of pan14-3-3 was also stronger in mitotic cells than the cells in interphase. As pEGFR localized mostly in the PM following EGF stimulation for 5 min, and in the endosome and the lysosome following longer EGFR stimulation, pEGFR is a good maker for the endocytic pathway. As shown in Figure 3E, 14-3-3 had limited co-localization with pEGFR at the PM and in the endosomes. 

Together, pan14-3-3 was localized to many compartments of the cell, including actin fibers, microtubules, mitochondria, the ER, the PM, the nucleus, and the cytosol.

### 2.3. Subcellular Localization and Translocation of 14-3-3β in Response to EGF and During the Cell Cycle

**Figure 4:**
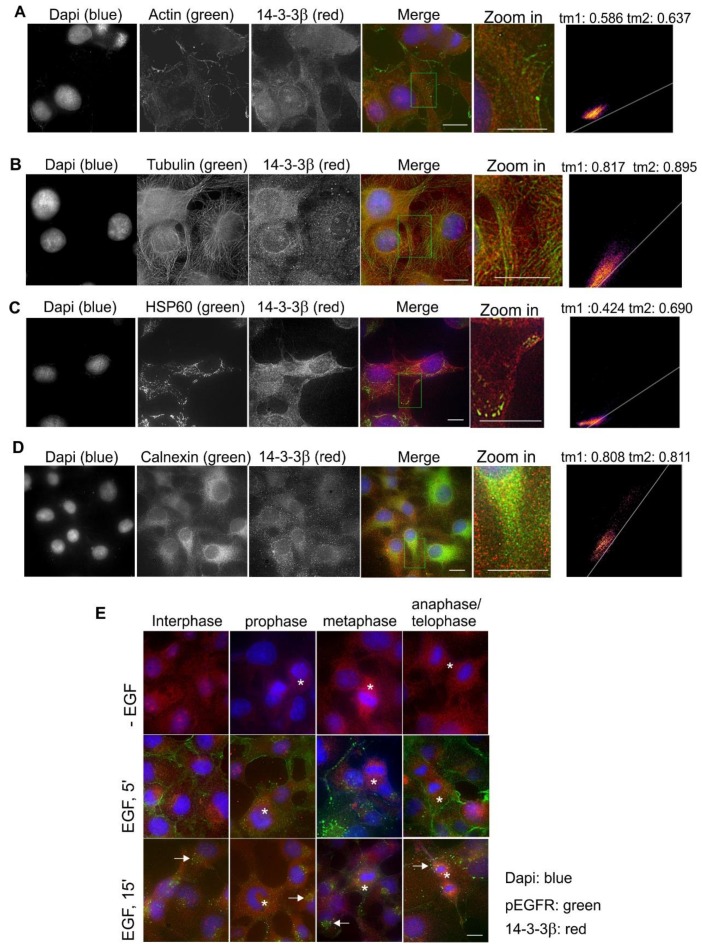
Subcellular localization of 14-3-3β in Cos-7 cells by double indirect immunofluorescence. (**A**) Co-localization of 14-3-3β (red) and actin (green); (**B**) co-localization of 14-3-3β (red) and tubulin (green); (**C**) co-localization of 14-3-3β (red) and HSP60 (green); (**D**) co-localization of 14-3-3β (red) and calnexin (green). For (**A**–**D**), the zoomed-in areas were used to calculate Mander’s coefficients. (**E**) Subcellular localization of 14-3-3β during the cell cycle and in response to EGF. With or without EGF stimulation, 14-3-3β (red) and pEGFR (green) were revealed by double indirect immunofluorescence. Mitotic cells are labeled by * and endosomes are marked by arrows. Scale bar = 10 μm.

We then examined the subcellular localization and the translocation of 14-3-3β in response to EGF and during the cell cycle in Cos-7 cells. Co-localization by double indirect immunofluorescence showed that 14-3-3β had some co-localization with actin fibers (Figure 4A), but quite strong co-localization with microtubules (Figure 4B). We also observed co-localization of 14-3-3β with HSP60 in the distal region of the cell (Figure 4C) and with calnexin (Figure 4D). 

We did not observe meaningful changes in terms of the subcellular localization of 14-3-3β in response to EGF stimulation (Figure 4E). The 14-3-3β stain for mitotic cells was stronger than that in interphase cells, but this could be due to the round-up of the cell during mitosis (Figure 4E). We also did not observe the co-localization between 14-3-3β and pEGFR (Figure 4E). 

### 2.4. Subcellular Localization and Translocation of 14-3-3ε in Response to EGF and During the Cell Cycle

**Figure 5:**
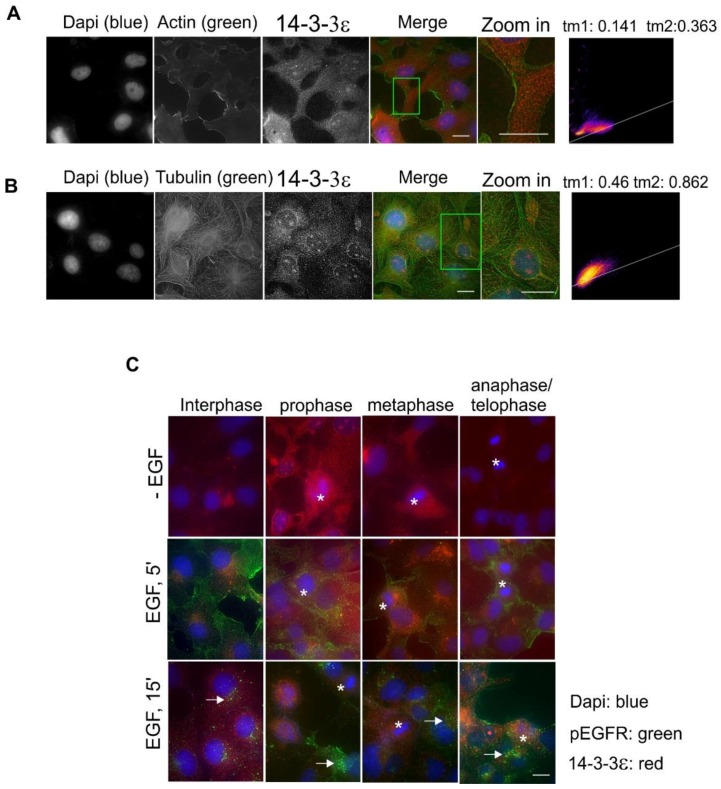
Subcellular localization of 14-3-3ε in Cos-7 cells by double indirect immunofluorescence. (**A**) Co-localization of 14-3-3ε (red) and actin (green); (**B**) co-localization of 14-3-3ε (red) and tubulin (green). For (**A**,**B**), the zoomed-in areas were used to calculate Mander’s coefficients. (**C**) Subcellular localization of 14-3-3ε during the cell cycle and in response to EGF. With or without EGF stimulation, 14-3-3ε (red) and pEGFR (green) were revealed by double indirect immunofluorescence. Mitotic cells are labeled by * and endosomes are marked by arrows. Scale bar = 10 μm.

We next examined the subcellular localization and the translocation of 14-3-3ε in response to EGF and during the cell cycle in Cos-7 cells. Co-localization by double indirect immunofluorescence showed that 14-3-3ε had very weak co-localization with actin fibers (Figure 5A), but strong co-localization with microtubules (Figure 5B). 14-3-3ε is most diffusely distributed within the cytoplasm.

EGF stimulation did not cause observable changes in the subcellular localization of 14-3-3ε (Figure 5C). There were also very few changes in 14-3-3ε subcellular distributions during mitosis (Figure 5C). We also did not observe co-localization between 14-3-3ε and pEGFR (Figure 5C).

### 2.5. Subcellular Localization and Translocation of 14-3-3ƞ in Response to EGF and During the Cell Cycle

**Figure 6:**
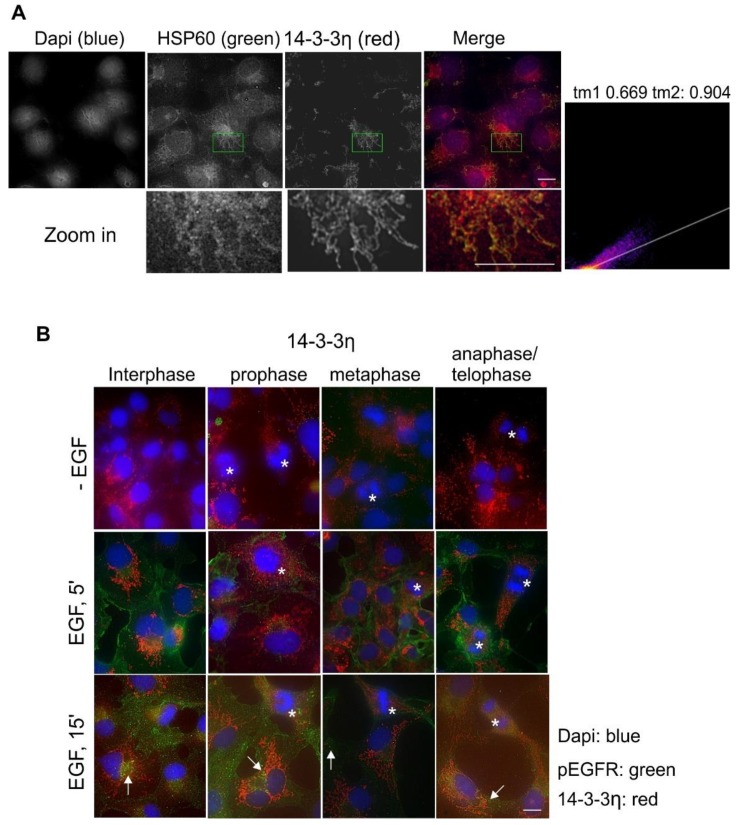
Subcellular localization of 14-3-3η in Cos-7 cells by double indirect immunofluorescence. (**A**) Co-localization of 14-3-3η (red) and HSP60 (green). The zoomed-in areas were used to calculate Mander’s coefficients. (**B**) Subcellular localization of 14-3-3η during the cell cycle and in response to EGF. With or without EGF stimulation, 14-3-3η (red) and pEGFR (green) were revealed by double indirect immunofluorescence. Mitotic cells are labeled by * and endosomes are marked by arrows. Scale bar = 10 μm.

The next isoform studied was 14-3-3ƞ. By using double indirect immunofluorescence, we first examined the co-localization of 14-3-3ƞ with HSP60, as 14-3-3ƞ showed very clear and almost exclusive mitochondrial localization in Figure 1. Indeed, as shown in Figure 6A, 14-3-3ƞ very strongly co-localized with HSP60. Moreover, EGF stimulation did not change the mitochondrial localization of 14-3-3ƞ (Figure 6B). However, in most cases, the stain of 14-3-3ƞ was weaker during mitosis (Figure 6B).

### 2.6. Subcellular Localization and Translocation of 14-3-3γ in Response to EGF and During the Cell Cycle

**Figure 7:**
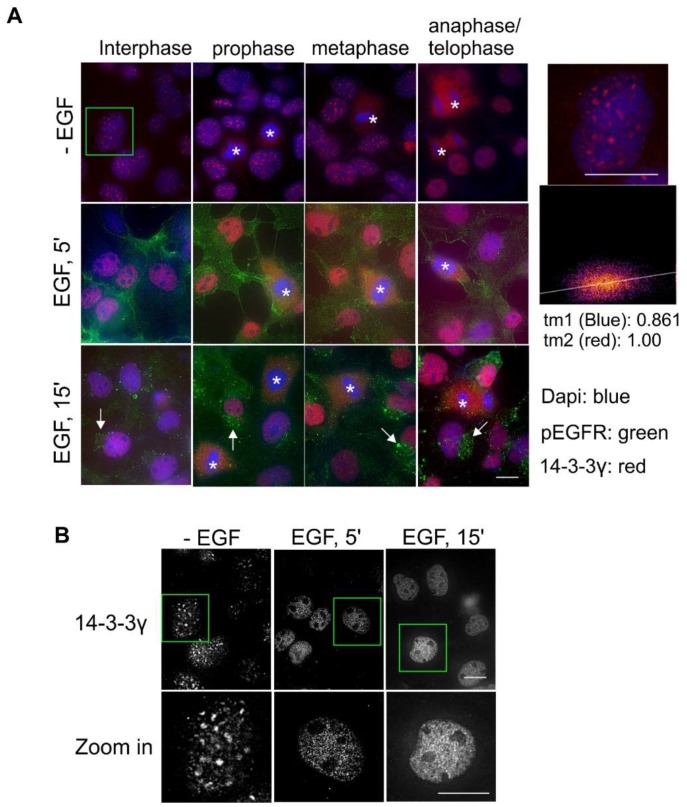
Subcellular localization of 14-3-3γ in Cos-7 cells by double indirect immunofluorescence. (**A**) Subcellular localization of 14-3-3γ during the cell cycle and in response to EGF. With or without EGF stimulation, 14-3-3γ (red) and pEGFR (green) were revealed by double indirect immunofluorescence. Mitotic cells are labeled by * and endosomes are marked by arrows. The zoomed-in areas were used to calculate Mander’s coefficients between blue and red channels. (**B**) Black and white images highlight the changes in the 14-3-3γ stain. The three images are the same as the three images in the far left panel in (A). Scale bar = 10 μm.

We showed above that 14-3-3γ was exclusively localized to the nucleus (Figure 1). We examined the translocation of 14-3-3γ in response to EGF and during the cell cycle by double indirect immunofluorescence in Cos-7 cells. As shown in Figure 7A, with or without EGF stimulation, 14-3-3γ is only localized to the nucleus. However, it is interesting to notice that following EGF stimulation, the pattern of 14-3-3γ stain within the nucleus changes. In the absence of EGF, 14-3-3γ formed large particles in the nucleus; however, following EGF stimulation for 5 and 15 min, 14-3-3γ formed much smaller particles (Figure 7A,B). During mitosis, 14-3-3γ is diffusely distributed throughout the cells as the nuclear membrane is broken down (Figure 1A). It seemed that 14-3-3γ was not associated with any structures but just evenly distributed in the cell.

### 2.7. Subcellular Localization and Translocation of 14-3-3σ in Response to EGF and During the Cell Cycle

**Figure 8:**
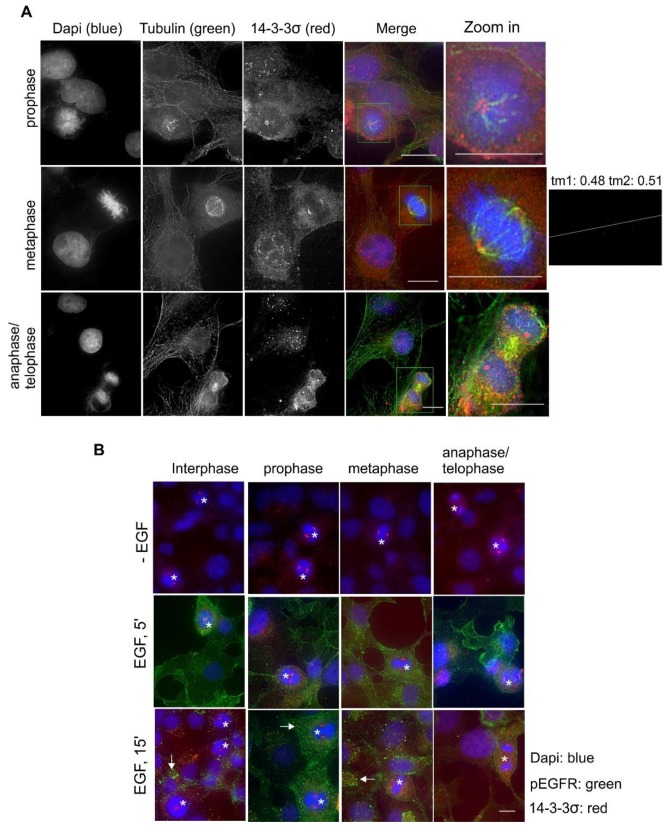
Subcellular localization of 14-3-3σ in Cos-7 cells by double indirect immunofluorescence. (**A**) Co-localization of 14-3-3σ (red) and tubulin (green) during mitosis. The zoomed-in areas were used to calculate Mander’s coefficients. (**B**) Subcellular localization of 14-3-3σ during the cell cycle and in response to EGF. With or without EGF stimulation, 14-3-3σ (red) and pEGFR (green) were revealed by double indirect immunofluorescence. Mitotic cells are labeled by * and endosomes are marked by arrows. Scale bar = 10 μm.

We next examined the subcellular localization and the translocation of 14-3-3σ in response to EGF and during the cell cycle in Cos-7 cells by double indirect immunofluorescence. We showed above that 14-3-3σ localized to both the cytoplasm and the nucleus in interphase but strongly associated with the putative centrosome during mitosis (Figure 1). Here, we first examined the co-localization of 14-3-3σ with microtubules during mitosis. As shown in Figure 8A, 14-3-3σ strongly co-localized with microtubules in the spindle pole of tubulin spindle. We also examined the effects of EGF on the subcellular localization of 14-3-3σ and found no changes in response to EGF (Figure 8B).

### 2.8. Subcellular Localization and Translocation of 14-3-3θ in response to EGF and During the Cell Cycle

**Figure 9:**
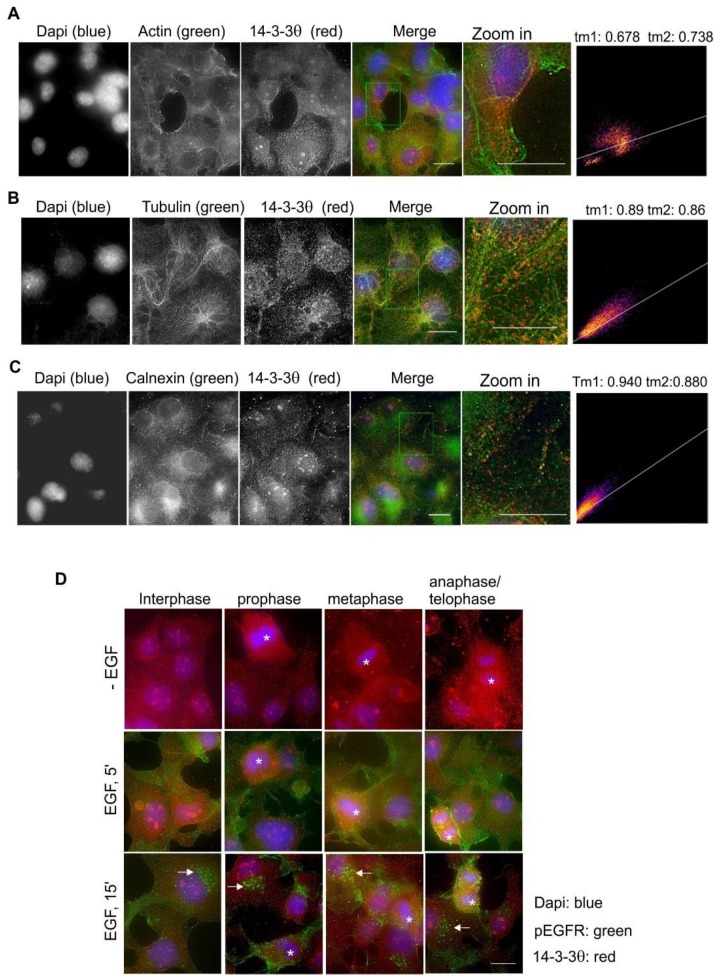
Subcellular localization of 14-3-3θ in Cos-7 cells by double indirect immunofluorescence. (**A**) Co-localization of 14-3-3θ (red) and actin (green); (**B**) co-localization of 14-3-3θ (red) and tubulin (green); (**C**) co-localization of 14-3-3θ (red) and calnexin (green). For (**A**–**C**), the zoomed-in areas were used to calculate Mander’s coefficients. (**D**) Subcellular localization of 14-3-3θ during the cell cycle and in response to EGF. With or without EGF stimulation, 14-3-3θ (red) and pEGFR (green) were revealed by double indirect immunofluorescence. Mitotic cells are labeled by * and endosomes are marked by arrows. Scale bar = 10 μm.

We examined the co-localization of 14-3-3θ with various markers by double indirect immunofluorescence in Cos-7 cells. 14-3-3θ was co-localized with actin near the PM (Figure 9A). There was also noticeable co-localization between 14-3-3θ and microtubules near the nuclear membrane (Figure 9B). There was a strong co-localization between 14-3-3θ and calnexin (Figure 9C). We then examined the translocation of 14-3-3θ in response to EGF and during the cell cycle. The addition of EGF did not change the subcellular localization of 14-3-3θ (Figure 9D). During mitosis, in the absence of nuclear membrane, 14-3-3θ is distributed throughout the cell and no specific pattern changes were observed (Figure 9D).

### 2.9. Subcellular Localization and Translocation of 14-3-3ζ in Response to EGF and During the Cell Cycle

**Figure 10:**
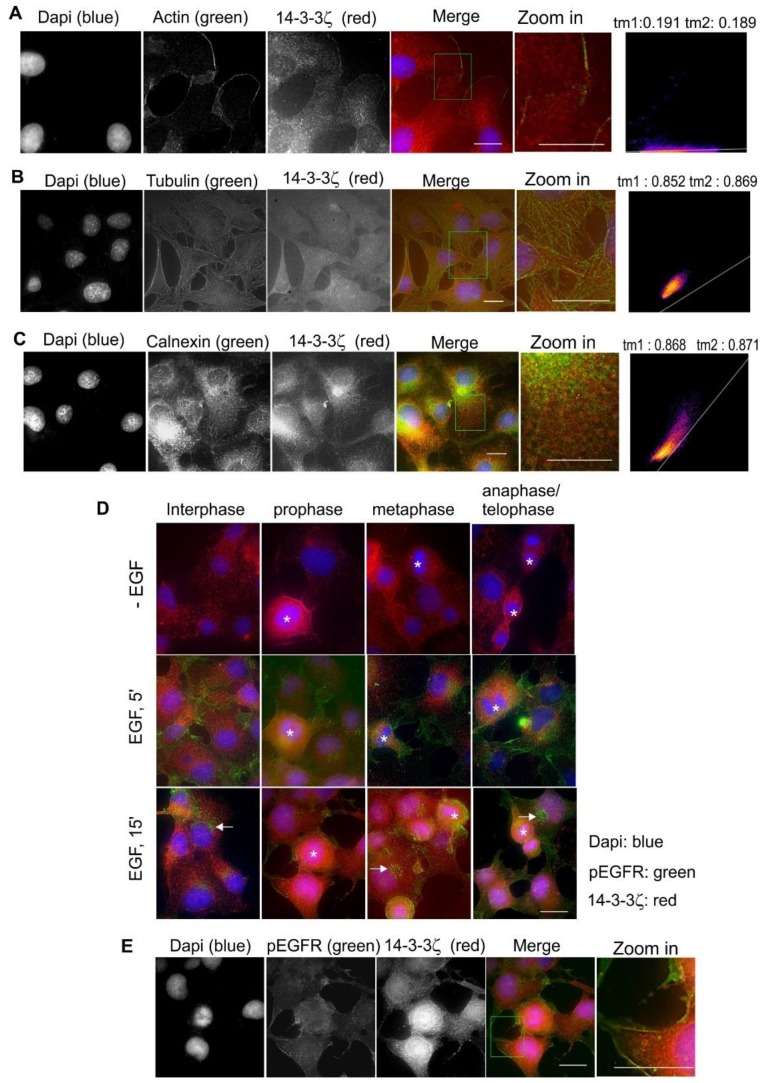
Subcellular localization of 14-3-3ζ in Cos-7 cells by double indirect immunofluorescence. (**A**) Co-localization of 14-3-3ζ (red) and actin (green); (**B**) co-localization of 14-3-3ζ (red) and tubulin (green); (**C**) co-localization of 14-3-3ζ (red) and calnexin (green). (**D**) Subcellular localization of 14-3-3ζ during the cell cycle and in response to EGF. With or without EGF stimulation, 14-3-3ζ (red) and pEGFR (green) were revealed by double indirect immunofluorescence. (**E**) Co-localization of 14-3-3ζ (red) and pEGFR (green) following EGF stimulation for 5 min. Mitotic cells are labeled by * and endosomes are marked by arrows. Scale bar = 10 μm.

Finally, we examined the co-localization of 14-3-3ζ with actin, tubulin, and calnexin. As shown in Figure 10A, 14-3-3ζ showed strong co-localization with actin fibers near the PM. 14-3-3ζ also showed co-localization with microtubules (Figure 10B). The co-localization between 14-3-3ζ and calnexin was also observed (Figure 10C). Stimulation with EGF did not induce the subcellular translocation of 14-3-3ζ; however, the fiber-like pattern of 14-3-3ζ seemed stronger in response to EGF (Figure 10D). 14-3-3ζ, in mitotic cells, generally had stronger PM localization (Figure 10D). Interestingly, we observed the co-localization between 14-3-3ζ with pEGFR in the PM following EGF stimulation for 5 min (Figure 10E).

### 2.10. The Expression and Subcellular Localization of Total 14-3-3 Protein and Each 14-3-3 Isoform in Human Cell Lines

All of the above experiments were performed in Cos-7 cells derived from monkey kidney fibroblasts. Here, we further examined the subcellular localization of total 14-3-3 protein and each 14-3-3 isoform in human cell lines, including HEK 293 cells derived from human embryonic kidneys, and two breast cancer cell lines: MDA-MB-231 cells and MCF-7 cells.

**Figure 11:**
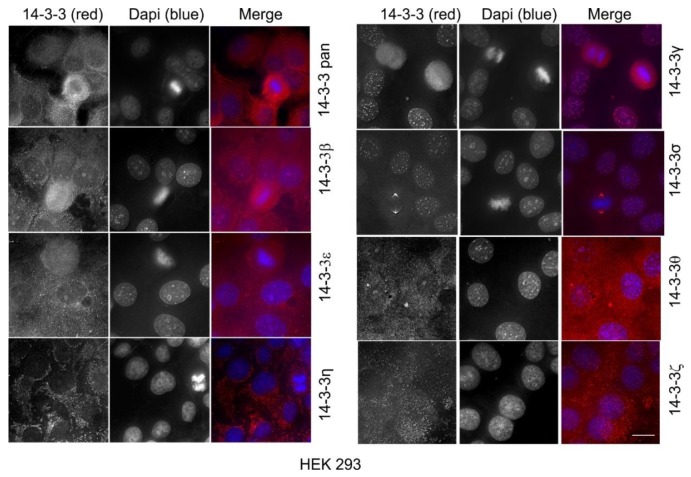
Subcellular localization of the total 14-3-3 protein and the seven 14-3-3 isoforms in HEK 293 cells by immunofluorescence. The total 14-3-3 protein was determined by a pan14-3-3 antibody. Each 14-3-3 isoform was determined by antibodies to each specific isoform. Immunoblotting, immunofluorescence, and subcellular fractionation were performed as described in Materials and Methods. 14-3-3 proteins were revealed by TRITC (red) and the chromatin was stained by DAPI (blue). Scale bar = 10 µm.

As shown in Figure 11, in HEK 293 cells, a total 14-3-3 protein was localized throughout the cells with the 14-3-3pan antibody. Like in Cos-7 cells, the most prominent cytoplasmic stain of pan 14-3-3 was the fiber-like pattern both near the plasma membrane and across the cell. One noticeable difference was that in Cos-7 cells, the fiber-like stain was mostly near the plasma membrane, but in HEK 293 cells, the fiber-like stain was mostly within the cytoplasm. Like in Cos-7 cells, 14-3-3β and ε localized to both the nucleus and the cytoplasm and did not associate with specific organelles. We also observed very specific and strong staining patterns for 14-3-3ƞ, γ, and σ. Similar Cos-7 cells, in HEK 293 cells, 14-3-3ƞ showed a strong mitochondrial pattern. 14-3-3γ was mostly localized to the nucleus. In interphase cells, the 14-3-3σ stain was stronger in the nucleus than in the cytoplasm. However, most strikingly, 14-3-3σ showed strong and specific putative centrosome staining during mitosis. 14-3-3θ and ζ showed no specific staining patterns and seemed localized throughout the cell.

**Figure 12:**
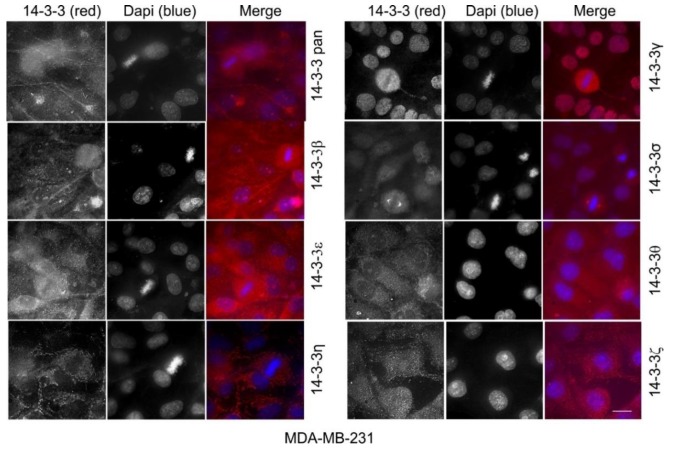
Subcellular localization of the total 14-3-3 protein and the seven 14-3-3 isoforms in MDA-MB-231 cells by immunofluorescence. The total 14-3-3 proteins were determined by a pan14-3-3 antibody. Each 14-3-3 isoform was determined by antibodies to each specific isoform. Immunoblotting, immunofluorescence, and subcellular fractionation were performed as described in Materials and Methods. 14-3-3 proteins were revealed by TRITC (red) and the chromatin was stained by DAPI (blue). Scale bar = 10 µm.

**Figure 13:**
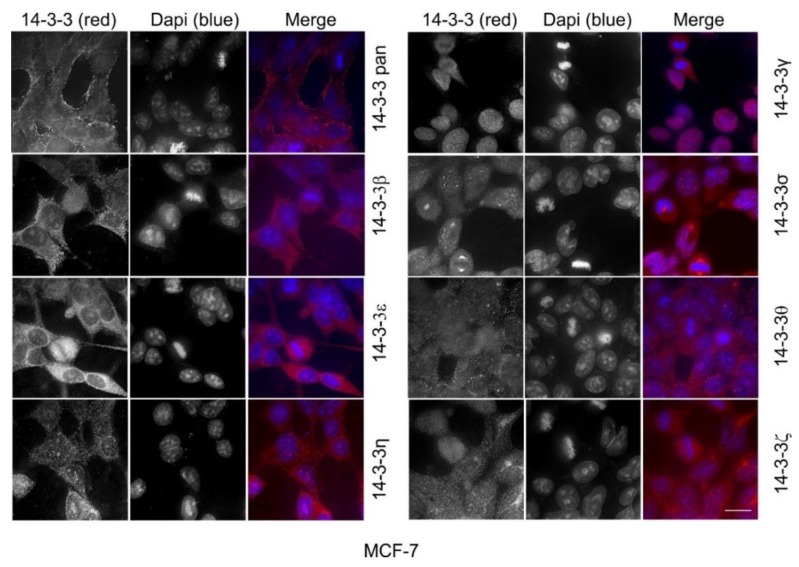
Subcellular localization of the total 14-3-3 protein and the seven 14-3-3 isoforms in MCF-7 cells by immunofluorescence. The total 14-3-3 proteins were determined by a pan14-3-3 antibody. Each 14-3-3 isoform was determined by antibodies to each specific isoform. Immunoblotting, immunofluorescence, and subcellular fractionation were performed as described in Materials and Methods. 14-3-3 proteins were revealed by TRITC (red) and the chromatin was stained by DAPI (blue). Scale bar = 10 µm.

We then examined the subcellular localization of the total 14-3-3 protein and each 14-3-3 isoform in human breast cancer cell lines, including MDA-MB-231 cells (Figure 12) and MCF-7 cells (Figure 13). As shown in Figure 12 and Figure 13, 14-3-3pan and each of the seven isoforms showed very similar staining patterns to Cos-7 cells and to HEK 293 cells. While 14-3-3β, ε, θ, and ζ did not specifically associate with any organelle, 14-3-3ƞ, γ, and σ clearly associated with the mitochondria, the nucleus, and the centrosome, respectively.

## 3. Discussion

In this research, we extensively examined the subcellular localization of the total 14-3-3 protein and each of the 14-3-3 isoforms in different mammalian cells with quite a range of origins. We found that 14-3-3 proteins are widely distributed throughout the cell and associated with many subcellular structures/organelles, including the PM, mitochondria, the ER, the nucleus, microtubules, and actin fibers. This broad distribution underlines the multiple functions identified for 14-3-3 proteins. We also observed EGF-stimulated translocation of some 14-3-3 isoforms and the redistribution of some 14-3-3 isoforms during mitosis. 

Our results reveal both similarities and differences in the subcellular localization among these seven 14-3-3 isoforms. This could be partially related to their sequence similarities and differences. Each of the seven 14-3-3 protein isoforms is encoded by a separate gene and the differences among the isoforms are mostly in the short, variable stretches of the primary structure [40,41]. Phylogenetic analysis indicates that the seven isoforms can be grouped into three pairs, including β and ζ, σ and θ, and ƞ and γ, with ε being unpaired. The two isoforms within each pair are most similar to each other [41,42]. We did observe similar subcellular localization between 14-3-3β and ζ. They both localize to the cytoskeleton, ER, and nucleus (Figure 4 and Figure 10). Both 14-3-3σ and θ are also strongly associated with microtubules, but σ is primarily associated with centrosomes and the microtubule spindle during mitosis (Figure 8 and Figure 9). However, the subcellular localizations of 14-3-3ƞ and γ are quite different. 14-3-3γ is almost exclusively in the nucleus and 14-3-3ƞ is almost exclusively in the mitochondria (Figure 6 and Figure 7).

Immunofluorescence imaging is the major method employed in this research to study the colocalization of the 14-3-3 protein isoforms with various marks for the subcellular compartments. Besides observing the colocalization by the overlapping of the colors, we also quantitatively analyzed the colocalization by measuring the Mander’s coefficients. It is a very complicated task to quantitate the co-localization, and several methods have been used; each has its advantage and disadvantage [43]. Manders’ method determines the overlap of two images while taking into account the pixel intensity. 

To determine if our observations in Cos-7 cells were applicable to other cells, we performed the same indirect immunofluorescence experiments in human cell lines, including HEK 293 cells derived from human embryonic kidneys, and two breast cancer cell lines: MDA-MB-231 cells and MCF-7 cells (Figure 11, Figure 12 and Figure 13). We showed that the subcellular localization of 14-3-3 proteins was very similar in these human cells as in Cos-7 cells. The most interesting and significant observations from all of these cell lines are the localizations of 14-3-3η to the mitochondria, 14-3-3γ to the nucleus, and 14-3-3σ to the centrosome during mitosis.

One of the major functions of 14-3-3 proteins is to regulate the remodeling of the cytoskeleton and cell migration [41]. We show in this study that 14-3-3 proteins are strongly associated with the cytoskeleton, including both actin fibers and microtubules. Both pan14-3-3 and 14-3-3ζ show strong actin fiber associations (Figure 1, Figure 3 and Figure 10), both 14-3-3β and ε also show weak actin fiber associations (Figure 4 and Figure 5). These results suggest that 14-3-3 proteins may act to regulate actin fiber formation. Indeed, it was initially suggested that 14-3-3 proteins directly interact with F-actin; however, later research suggested that 14-3-3 proteins regulate actin fiber formation through the regulation of cofilin [41,44]. The 14-3-3 isoforms reported to regulate F-actin are 14-3-3γ and ζ [41,45,46]. A growing number of proteins involved in actin remodeling have recently been identified as 14-3-3 binding partners [5,6,7,9,47].

We also showed that 14-3-3β, ε, θ, and ζ all co-localize with microtubules, suggesting their roles in the regulation of microtubule formation. Our findings are mostly consistent with previous reports. Indeed, it is reported that 14-3-3ε, η, σ, γ, θ, and ζ regulate the formation and dynamic nature of microtubules [48,49,50,51,52]. It is generally believed that 14-3-3 proteins regulate microtubules through tau proteins that stabilize microtubules [38,41]. Many studies also implicate small GTPases, such as Rac and RhoA, in the regulation of microtubules by 14-3-3 proteins [53,54]. However, as 14-3-3β, ε, θ, and ζ were all shown with strong localization cross the cytoplasm, the specificity of their co-localization with microtubules needs to be confirmed by further research.

One interesting observation in this study was the prominent localization of 14-3-3ƞ in the mitochondria (Figure 6). We also observed the weak localization of 14-3-3β in the mitochondria (Figure 4). 14-3-3 proteins have been reported to localize to the mitochondria and to regulate various functions related to the mitochondria, including cell apoptosis and oxidation [4,55,56]. Two particular isoforms, 14-3-3ƞ and γ, are implicated in mitochondrial function. It has been reported that 14-3-3ƞ protects against mitochondria-mediated apoptosis [56]. 14-3-3ƞ is also involved in the transportation and apoptosis-related function of Bcl-2 in the mitochondria [57,58]. Inconsistent with these reported functions of 14-3-3ƞ in apoptosis, we did show that 14-3-3η is almost exclusively localized to the mitochondria (Figure 6). While 14-3-3γ is also implicated in mitochondrial function by some research [59,60], we did not observe any mitochondrial localization of 14-3-3γ (Figure 7).

Another interesting finding was the prominent and specific localization of 14-3-3σ to the centrosome during mitosis (Figure 8). 14-3-3 protein has been implicated in centrosome regulation [33,34,61]. It was initially reported that 14-3-3ε and γ localize to the centrosome and the mitotic spindle apparatus in mouse leukemic FDCP cells [33]. It was shown recently that 14-3-3 protein regulates the formation of centriolar satellites by sequestering CEP131 [61]. In a detailed study, 14-3-3γ localized to the centrosome and the loss of 14-3-3γ led to centrosome amplification [34]. However, our findings suggest that 14-3-3γ is exclusively localized to the nucleus in interphase cells, but diffusely distributed throughout the cell during mitosis when the nuclear membrane is broken down (Figure 7). We found that 14-3-3σ is localized to both the nucleus and the cytoplasm in interphase, but prominently associated with the centrosome during mitosis (Figure 8). Our observations regarding 14-3-3γ and σ in Cos-7 cells were confirmed in the other three cell lines, including HEK 293, MDA-MB-231, and MCF-7 cells (Figure 11, Figure 12 and Figure 13). Centrosomes are the major microtubule nucleating and organizing centers and are critical for the proper formation and dynamics of the microtubule spindle during mitosis. Thus, the centrosome localization of 14-3-3σ suggests its critical role in regulating the microtubule spindle and mitosis. 14-3-3 proteins have been shown to interact with multiple proteins involved in mitotic regulation [4]. It is interesting to notice that 14-3-3σ is downregulated in many tumor types, suggesting tumor suppressor activity [54]. Thus, 14-3-3σ could be a target for cancer therapy. 

The mitotic centrosome localization of 14-3-3σ is the most prominent change observed in terms of the cell cycle regulation of 14-3-3 proteins. The other effects of the cell cycle on the subcellular distribution of the 14-3-3 proteins are to break down the nuclear membrane to allow the potential interaction among the different isoforms. It is well established that 14-3-3 proteins function by forming homo- and/or heterodimers [1,62]. Although most 14-3-3 protein isoforms are co-localized in the cytosol and the nucleus, 14-3-3γ is only one localized to the nucleus. Thus, mitosis may be the only time when all the 14-3-3 isoforms are potentially able to interact with each other. 

We also studied the effects of EGF on the subcellular localization of 14-3-3 proteins. Binding to a client protein could re-locate 14-3-3 proteins and the binding of 14-3-3 proteins to a client protein is mostly dependent on the phosphorylation of the client protein [1]. Protein phosphorylation is mostly regulated by growth factor stimulation. Thus, it was interesting to determine whether the subcellular localization of 14-3-3 proteins is affected by growth factor stimulation. We identified two changes among the 14-3-3 protein isoforms in response to EGF. First, 14-3-3γ formed large particles in the nucleus in the absence of EGF. However, in response to EGF stimulation for 5 or 15 min, these large particles disappeared, and instead, many smaller particles appeared. This could be due to the dissociation of 14-3-3γ from the initial larger structures and the re-association with some smaller structures. Many nuclear structures and subdomains have been discovered, including nucleus speckles, Cajal bodies, promyelocytic leukemia nuclear bodies (PMLs), Polycomb bodies, and histone locus bodies (HLBs) [63]. To determine the nuclear function of 14-3-3γ, it would be interesting to determine which subnuclear structures 14-3-3γ is associated with before and after EGF stimulation. The other observation was the enhanced association of some 14-3-3 isoforms, including β, ε, σ, θ, and ζ, with actin fibers and microtubules. This is not surprising as EGF has been shown to regulate cytoskeleton remodeling and cell migration [64,65].

## 4. Conclusions

In conclusion, 14-3-3 proteins are broadly distributed throughout the cell and associated with many subcellular structures/organelles, including the PM, mitochondria, the ER, the nucleus, microtubules, and actin fibers. This broad distribution underlines the multiple functions identified for 14-3-3 proteins. The subcellular distributions of various 14-3-3 isoforms are summarized in Table 1. As shown in Table 1, the different isoforms of 14-3-3 proteins have distinctive subcellular localizations that suggest their distinctive cellular functions. While 14-3-3β, ε, θ, and ζ are not specifically associated with any organelle, 14-3-3ƞ, γ, and σ showed very specific and strong localizations to specific organelles. 14-3-3ƞ is almost exclusively localized to the mitochondria, 14-3-3γ is only localized to the nucleus, and 14-3-3σ strongly and specifically associated with the centrosome during mitosis.

## 5. Materials and Methods

### 5.1. Cell Culture and Treatment

Cos-7, HEK-293, MDA-MB-231, and MCF-7 cells were cultured in Dulbecco’s modified Eagle’s medium containing 10% FBS and supplemented with non-essential amino acids, and were maintained in a 5% CO_2_ atmosphere at 37 °C. For EGF stimulation, cells were starved in DMEM containing FBS 1% overnight, and then incubated with 50 ng/mL EGF (final concentration) in this starvation medium for the indicated time. 

### 5.2. Chemicals and Antibodies

Here is the information regarding the 14-3-3 antibodies used in this research. The antibodies used for the main research were as follows: seven monoclonal mouse antibodies were from Santa Cruz Biotechnology Inc. (SCBT) (Dallas, TX, USA), including 14-3-3pan (H-8) (sc-1657), 14-3-3β (A-6) (sc-25276), 14-3-3ε (F-3) (sc-393177), 14-3-3η (6A12) (sc-293464), 14-3-3γ (D-6) (sc-398423), 14-3-3σ (E-11) (sc-166473), and 14-3-3ζ (1B3) (sc-293415). 14-3-3θ was from Novus Biologicals Inc. (NBP 1-21301). The 14-3-3 antibodies used to confirm our results included the following: 14-3-3θ (5J20) (sc-69720) and 14-3-3ζ (G-2) (sc-518031) were from SCBT, and 14-3-3η (10847-MM06) was from Sino Biological Inc. Blocking peptides were also from SCBT. Rabbit polyclonal anti-lamin A, goat polyclonal anti-pEGFR1086 antibody was purchased from SCBT. Goat polyclonal antibodies against α-actin were from Sigma-Aldrich (St. Louis, MO, USA). Rabbit monoclonal anti-α tubulin was from Abcam (Toronto, ON, Canada). Rabbit polyclonal anti-HSP60 antibody was purchased from R&D Systems (Minneapolis, MN, USA).

### 5.3. Cell Lysates and Immunoblotting

To generate total cell lysates, the cells cultured in 10 cm plates were scraped, lysed, and homogenized in ice-cold Mammalian Protein Extraction Reagent (Pierce, Rockford, IL, USA) with a protease and phosphatase inhibitor cocktail (0.1 mM 4-(2-aminoethyl)-benzenesulfonyl fluoride, 10 µg/mL aprotinin, 1 µM pepstatin A, and 0.5 mM Na_3_VO_4_, 0.02% NaN_3_). The lysates were then centrifuged at 21,000× *g* at 4 °C for 15 min. The supernatant was collected, the protein concentration was quantified, and the sample was boiled in SDS-loading buffer for 5 min. Immunoblotting was performed as previously described [66].

### 5.4. Indirect Immunofluorescence

Cells were cultured on the glass coverslips for 48 h before treatment. After treatment, the cells were rinsed in Tris-buffered saline (TBS: 6% Tris, 8.8% NaCl, 85.2% dH_2_O, pH 7.6) and were fixed by −20 °C methanol for 5 min. Cells were permeabilized with TBS containing 0.2% Triton X-100 for 10 min; that was followed by blocking with TBS containing 1% BSA and 0.1% Triton X-100 for an hour. After blocking, the coverslips were incubated in 1 µg/mL primary antibody in TBS with 0.1% Triton X-100, as indicated, for an hour. Afterward, the coverslips were rinsed in TBS with 0.1% Triton X-100 three times each for 5 min and then incubated in 1 µg/mL solution of FITC- and/or TRITC-conjugated secondary antibody in TBS with 0.1% Triton X-100 for an hour in the dark. Thereafter, the coverslips were washed completely in TBS and incubated in 1 µg/mL of 4′,6-diamidino-2-phenylindole (DAPI) solution in TBS for 5 min at room temperature in the dark. The coverslips were then mounted on glass slides and observed using a GE Healthcare DeltaVision Deconvolution Microscope system (GE Healthcare Life Science, Mississauga, ON, Canada). All of the images were deconvolved.

### 5.5. Co-Localization Analysis of IF Images by Mander′s Overlap Coefficient

To quantitatively determine the colocalization, the IF images were analyzed and Mander′s overlap coefficients m1 and m2 on the specified ROI were calculated [43]. This measurement generates a value for each channel which is a modification of Mander’s original formula, except the thresholds that have been calculated are used. Then, data were represented as 2D histograms. For example, if the red-green pair of channels is selected and tM1 (red) and tM2 (green) are 0.5 and 0.7, respectively, this means that 50% red pixels colocalize with green pixels, but 70% of green pixels colocalize with red ones, For this quantification we used Coloc2 Plugin (Fiji) in ImageJ software (ImageJ2, NIH, Bethesda, MD, USA).

(1) $$\begin{array}{c} m_{1}=\frac{\sum_{i}S1_{i},\;coloc}{\sum_{i}S1_{i},}\\ m_{2}=\frac{\sum_{i}S2_{i},\;coloc}{\sum_{i}S2_{i},} \end{array},$$

where *S*1*_i_,_coloc_* = *S*1*_i_* if *S*2*_i_* > 0 and *S*2*_i_,_coloc_* = *S*2*_i_* if *S*1*_i_* > 0.

Usually, Mander’s value is expressed as a value between 0.0 and 1.0. In general, a value between 0.8–1.0 is considered a very strong colocalization. A value between 0.60 and 0.8 is considered a strong colocalization. A value between 0.4 and 0.6 is considered a moderate colocalization. A value between 0.2 and 0.4 is considered a weak colocalization. A value between 0 and 0.2 is considered a negative colocalization. However, it always depends on the purpose of the biological analysis and how we define colocalization significance in our study. Data are represented in Tm1, Tm2, and 2D histograms. For all histograms, the y-axis is the green channel and the x-axis is the red channel (14-3-3 protein) except for Figure 7. In Figure 7, the y-axis is the blue channel and the x-axis is the red channel.

### 5.6. Subcellular Fractionation

Cos-7 cells in 10 cm plates were scraped into homogenization buffer (0.25 M sucrose, 20 mM Tris-HCl, pH 7.0, 1 mM MgCl_2_, 4 mM NaF, 0.5 mM Na_3_VO_4_, 0.1 mM 4-(2-aminoethyl)-benzenesulfonyl fluoride, 10 μg/mL aprotinin, and 1 μM pepstatin A). Each 10-cm plate was scraped into 500 µL of homogenization buffer. The cells were then lysed using a 27 Gauge syringe for 15 times and were left on ice for 30 min. Afterward, the cell homogenates were centrifuged at 3000 rpm for 6 min. The resulting supernatant contained the cytoplasmic fraction and the pellet contained the nuclei. The supernatant was transferred into another tube and the pellet was washed with 500 µL homogenization buffer 3 times. The pellet was then dispersed using a micropipette and a 25 G syringe 10 times. Following the centrifugation of the pellet at 3000 rpm for 15 min, the supernatant was discarded. The pellet was re-suspended in TBS 0.1% SDS and sonicated briefly on a continuous setting for 3 s to shear genomic material and homogenize the lysate. 

### 5.7. siRNA

In a six-well tissue culture plate, 2 × 10^5^ cells were seeded per well in 2 mL antibiotic-free normal growth medium supplemented with FBS; the cells were incubated at 37 °C in a CO_2_ incubator until the cells were 60–80% confluent. For each transfection, 0.8 mL siRNA transfection medium was added to each tube containing the siRNA transfection reagent mixture (Solution A + Solution B), and then the mixture was overlaid onto the washed cells. The cells were incubated 5–7 h at 37 °C in a CO_2_ incubator with 1 mL of normal growth medium containing two times the normal serum and antibiotic concentration (2 × normal growth medium) without removing the transfection mixture. Then, the cells were incubated for an additional 18–24 h. The medium was aspirated and replaced with fresh 1 × normal growth medium. The cells ere assayed by both immunoblotting and immunofluorescence 48 h after the addition of fresh medium.

### 5.8. Statistical Analysis

All protein bands were quantitated by densitometry using ImageJ software (ImageJ2, NIH, Bethesda, MD, USA). Data were statistically analyzed by one-way analysis of variance (ANOVA) using Prism V.8 software (GraphPad Software, La Jolla, CA, USA). Data are presented as means and standard deviations. *p* < 0.05 “APA” “American Psychological Association “was considered statistically significant.

## Figures and Tables

**Table 1:** The summary of the subcellular localizations of seven 14-3-3 isoforms.

14-3-3 isoform	Subcellular Localization
Beta	Cytoplasmic, ER and Mitochondria
Zeta	Cytoplasmic and Nuclear
Gamma	Nucleus
Epsilon	Cytoplasmic
Eta	Mitochondria
Tau/Theta	Cytoplasmic and ER
Sigma	Centrosome
